# Qianggan extract improved nonalcoholic steatohepatitis by modulating lncRNA/circRNA immune ceRNA networks

**DOI:** 10.1186/s12906-019-2577-6

**Published:** 2019-07-03

**Authors:** Mingzhe Zhu, Meng Li, Wenjun Zhou, Yang Yang, Fenghua Li, Li Zhang, Guang Ji

**Affiliations:** 1 Institute of Digestive Diseases, Longhua Hospital, Shanghai University of Traditional Chinese Medicine, No.725 South Wanping Road, Shanghai, 200032 China; 2 School of Public Health, Shanghai University of Traditional Chinese Medicine, Shanghai, 201203 China; 3 Experiment Center for Science and Technology, Shanghai University of Traditional Chinese Medicine, Shanghai, 201203 China

**Keywords:** Non-alcoholicsteatohepatitis, lncRNA, circRNA, ceRNA, Microarray, Bioinformatics

## Abstract

**Background:**

The traditional Chinese medicine prescription, Qianggan formula have been confirmed to be effective on non-alcoholic steatohepatitis (NASH), however, the underlying molecular mechanisms remain obscure.

**Methods:**

Thirty-six male C57BL/6 mice were randomly divided into three groups: normal chow diet group; methionine-and-choline-deficient diet (MCD) group, and Qianggan extract (QG) intervention group (0.4 g/kg daily) that fed with MCD. The efficacy of QG was biochemically and histologically evaluated. The expression profiles of messenger ribonucleic acids (mRNAs), long non-coding RNAs (lncRNAs) and circular RNAs (circRNAs) were examined using microarray and verified by RT-qPCR.

**Results:**

QG significantly improved the phenotypic characteristics of NASH, as serum alanine aminotransferase (ALT), aspartate aminotransferase (AST), alkaline phosphatase (ALP), and lactate dehydrogenase (LDH) levels and liver inflammatory cytokines were significantly decreased. By the cutoff of a 1.5-fold change and *P* < 0.05, 6193 mRNAs, 5692 lncRNAs and 4843 circRNAs were identified as differentially expressed between the MCD and normal groups, and 514 mRNAs, 1182 lncRNAs and 443 circRNAs were identified as differentially expressed between the QG and MCD groups. The intersections (244 mRNAs, 259 lncRNAs and 98 circRNAs) among the three groups were chosen for analysis. Gene Ontology (GO) terms and Kyoto Encyclopedia of Genes and Genomes (KEGG) pathway enrichment revealed that most overlapping mRNAs were related to immune functions such as natural-killer-cell-mediated cytotoxicity, intestinal immune network for IgA production, and T cell receptor signaling pathway. Pathway interactions, protein-protein interactions and molecular complex detection (MCODE) analysis identified numerous immune-related hub genes e.g. natural cytotoxicity triggering receptor 1(Ncr1), C-X-C motif chemokine ligand 9 (Cxcl9), Klra1, and Cd28. Finally, two lncRNAs (Sngh1 and Slc36a3os) and four circRNAs (circ_0009029, circ_0004572, circ_0009212 and circ_0009453) in competing endogenous RNA (ceRNA) networks were constructed by Cytoscape, and immune-related mRNAs (e.g., Cd28, Cd8a, Il15, and Klrk1) were involved in the ceRNA networks.

**Conclusions:**

LncRNA and circRNA-associated immune ceRNA networks might be the targets of QG in alleviating NASH, and our work may provide valuable clues for exploring the mechanisms underlying the effect of QG.

**Electronic supplementary material:**

The online version of this article (10.1186/s12906-019-2577-6) contains supplementary material, which is available to authorized users.

## Background

Nonalcoholic fatty liver disease (NAFLD) refers to a broad spectrum of diseases characterized by fat infiltration in the liver [1]. Non-alcoholic steatohepatitis (NASH) is a progressive form of NAFLD characterized by lobular inflammation, hepatocellular ballooning, and fibrosis with an inherent risk of progression to end-stage hepatocellular carcinoma [2, 3]. Twenty percent of NASH patients are reported to develop cirrhosis, and 30–40% of patients with NASH cirrhosis die from liver-related diseases, NASH has become the third most common indication for liver transplantation in the United States [4].

The pathogenesis of NASH has not been fully elucidated, and the mechanisms appear to be multifactorial. “Two hits” and “multiple hits” hypotheses have been proposed to describe the pathogenesis of NASH [5, 6]. Insulin resistance, inflammatory cytokines, and oxidative stress are thought to be important in the progression of NASH. Pharmacological agents such as insulin sensitizers, antioxidants, and lipid-lowering drugs have been implicated to improve NASH characteristics and progression [7]. However, there is no consensus regarding the effective and appropriate drug therapy for NASH.

Accumulating evidence suggests that traditional Chinese medicine formulae are promising agents for treating NASH [8]. The Qianggan formula was designed according to the theory of traditional Chinese medicine, contains 16 ingredients: *Artemisia scoparia* Waldst. & Kitam., *Isatis tinctoria* L., *Angelica sinensis*(Oliv.)Diels., *Paeonia lactiflora* Pall., *Salvia miltiorrhiza* Bunge., *Curcuma wenyujin* Y.H.Chen et C.Ling, *Astragalus membranaceus*(Fisch.)Bunge., *Codonopsis pilosula*(Franch.)Nannf., *Alisma orientale*(Sam.)Juz., *Polygonatum kingianum* Collett & Hemsl., *Rehmannia glutinosa* (Gaertn.) DC., *Dioscorea oppositifolia* L., *Crataegus pinnatifida* Bunge., *Medicated Leaven Massa Medicata Fermentata****,*** *Gentiana macrophylla* Pall., and *Glycyrrhiza uralensis* Fisch., and has been reported as an effective and safe remedy for the treatment of NAFLD/NASH [9]. Protecting hepatocytes is proposed; however, the molecular mechanisms underlying the efficacy are still unclear.

Coding ribonucleic acids (RNAs) coupled with non-coding (nc) RNAs are involved in NASH pathogenesis [10]. MicroRNAs (miRNAs), long non-coding RNAs (lncRNAs) and circular RNAs (circRNAs) are different species of ncRNAs [11]. MiRNAs are 18–25 nucleotides long, evolutionarily conserved, single-stranded RNA, that negatively modulate the expression of their target mRNAs [12]. Numerous miRNAs have been reported to be correlated with NASH [13, 14]. LncRNAs are RNA transcripts > 200 nucleotides, that can regulate gene expression through many patterns, including chromosome remodeling, and transcriptional and post-transcriptional processing. LncRNAs are involved in the pathogenesis of liver diseases and have potential diagnostic, prognostic, and therapeutic importance [15], and contribute to the development of NASH and fibrosis [10, 16]. LncRNAs can act as competing endogenous RNAs (ceRNAs) or miRNA sponges, interacting with miRNAs in a manner that sequesters these molecules and reduce their regulatory effect on target mRNAs [17]. Similar to lncRNAs, circRNAs (a class of RNA molecules that lack 5′-3′ ends and poly A tails and covalently form closed loops) can also function as ceRNAs or miRNA sponges [18]. The importance of ceRNAs has been addressed in various diseases. A deep RNA sequencing study constructed comprehensive circRNA-associated ceRNA networks, and inferred the diagnosis and therapy of Alzheimer’s disease [19]. Another study provided a lncRNA-related ceRNA network and suggested two-lncRNA signatures as independent biomarkers for the prognosis of lung squamous cell carcinoma [20].

To systemically understand the efficacy of Qianggan extract (QG) on NASH, a comprehensive microarray analysis of mRNAs, lncRNAs and circRNAs was conducted to obtain expression profiles. By identifying differentially expressed mRNAs, lncRNAs and circRNAs and constructing ceRNA networks, our data shed new light on potential novel biomarkers and drug targets of NASH.

## Methods

### Preparation of Qianggan extract

The Qianggan formula was composed of 16 ingredients (Table 1), and the extract was prepared using the following procedures: all of the ingredients were soaked in 10 times the volume of water and boiled for 2 h, the supernatant were collected, 8 times volume of water was added and the mixture was boiled another 1.5 h. Then, the supernatant was collected, 6 times the water volume was added, and the mixture was and boiled 1.5 h for the third time. All the supernatants were filtered, the PH was adjusted to 8. The extract powder of was then obtained by concentrating the solution to a density ratio of 1.35.

**Table 1:** The composition of Qianggan formula

Scientific name	Chinese Name	Pharmaceutical portion	Percent
*Artemisia scoparia* Waldst. & Kitam.	Yin-Chen	Cauline leaf	11%
*Isatis tinctoria* L.	Ban-Lan-Gen	Root	5.5%
*Angelica sinensis*(Oliv.)Diels	Dang-Gui	Root	5.5%
*Paeonia lactiflora* Pall.	Bai-Shao	Root	5.5%
*Salvia miltiorrhiza* Bunge.	Dan-Shen	Rhizome	11%
*Curcuma wenyujin* Y.H.Chen et C.Ling	Yu-Jin	Rhizome	5.5%
*Astragalus membranaceus*(Fisch.)Bunge	Huang-Qi	Root	11%
*Codonopsis pilosula*(Franch.)Nannf.	Dang-Shen	Root	5.5%
*Alisma orientale*(Sam.)Juz.	Ze-Xie	Tuber	5.5%
*Polygonatum kingianum* Collett & Hemsl	Huang-Jing	Rhizome	5.5%
*Rehmannia glutinosa* (Gaertn.) DC.	Shen-Di	Root	5.5%
*Dioscorea oppositifolia* L.	Shan-Yao	Rhizome	5.5%
*Crataegus pinnatifida* Bunge.	Shan-Zha	Fructus	4.4%
*Medicated Leaven Massa Medicata Fermentata*	Liu-Shen-Qu	Zymotic product	4.4%
*Gentiana macrophylla* Pall	Qin-Jiao	Root	4.4%
*Glycyrrhiza uralensis* Fisch.	Gan-Cao	Rhizome	4.4%

### Animal treatment and sample collection

Six-week-old male C57BL/6 mice were obtained from Shanghai Laboratory Animal Co. Ltd., and maintained in a specific pathogen-free environment with temperature-and humidity-controlled conditions. Thirty-six mice were allocated to six per cage, and randomly divided into three groups (*n* = 12 per group) based on bodyweight: the normal group, fed a chow diet; the methionine-and-choline-deficient diet (MCD) group, fed a MCD diet; and the QG group, fed a MCD diet and supplemented with the extract (Orient Pharmaceutical Co., Ltd., 0.4 g /kg daily). The extract was dissolved in distilled water and intragastrically administered (1 ml/100 g body weight) for 4 consecutive weeks, and the other mice were given an equal volume of distilled water. Body weight was recorded once per week. At the end of the experiment, mice were weighed at 8 am the next morning and anesthetized by intraperitoneal injection with 2% pentobarbital sodium (1.5 ml/kg bodyweight). Then, blood was collected by cardiac puncture, centrifuged at 3000 *rpm* for 10 min at 4 °C, and serum was separated. Liver tissue was quickly removed, rinsed with 0.9% sodium chloride solution and weighed. Liver tissues from the identical lobe and position were cut and then fixed in 10% neutral-buffered formalin. The remainder was stored at − 80 °C after snap freezing in liquid nitrogen. After sample collection, the animals were physically euthanized by quickly breaking the spines to avoid insufficient anesthetization, and bodies were disposed of by the central department of animal care.

All animal procedures were approved by the Animal Experiment Ethics Committee of Shanghai University of Traditional Chinese Medicine (approval number: 201703014).

### Determination of the serum enzymes levels

Serum alanine aminotransferase (ALT), aspartate aminotransferase (AST), alkaline phosphatase (ALP), and lactate dehydrogenase (LDH) were analyzed using the Hitachi full-automatic system with the corresponding kits (Wako, Richmond, VA, USA).

### Histology staining

Liver samples were fixed in 10% formalin for 48 h, embedded in paraffin, sectioned to 4 μm thickness, stained with hematoxylin and eosin (H&E), and examined under a light microscope at 200 × magnification.

### Microarray detection and analysis

#### RNA preparation and microarray detection

Liver total RNA was extracted using TaKaRa RNAiso Plus regent and RNA integrity was assessed using an Agilent Bioanalyzer 2100 (Agilent Technologies, Santa Clara, CA, US). RNA was further purified by an RNeasy mini kit (QIAGEN, Hilden, Germany) and RNase-Free DNase Set (QIAGEN), amplified and labeled by Low Input Quick Amp WT Labeling Kit (Agilent Technologies), purified and hybridized with a SBC-Mouse ceRNA microarray (SBC, Shanghai, China). Nine slides were scanned by an Agilent Microarray Scanner with default settings: dye channel, green; scan resolution, 3 μm; PMT 100%; 20 bit. Data were extracted with Feature Extraction version 10.7 (Agilent Technologies). Raw data were normalized by the Quantile algorithm, limma packages in R.

#### Microarray data processing

The statistical significance of differentially expressed mRNAs, lncRNAs and circRNAs among groups (MCD vs normal and QG vs MCD) was identified by the cutoff of 1.5-fold change and *P* < 0.05. The intersections of differentially expressed mRNAs, lncRNAs and circRNAs between MCD vs normal and QG vs MCD were determined by Venn diagrams. The overlapped mRNAs, lncRNAs and circRNAs were subjected to hierarchical clustering by Cluster version 3.0 to generate an overview of the characteristics of expression profiles.

#### Gene ontology (GO) and pathway analysis

To explore the main biological function of differentially expressed genes, functional enrichment was performed based on GO and Kyoto Encyclopedia of Genes and Genomes (KEGG) database [21, 22]. GO classifies gene functions according to molecular function, biological process and cellular component. Pathway enrichment analysis revealed the main metabolic and signaling pathways. The enriched pathways interaction networks were constructed using the Cytoscape plug-in ClueGO and CluePedia.

#### Protein-protein interaction (PPI) network analysis

PPI network analysis was performed based on the STRING database (https://string-db.org/). The network was visualized by Cytoscape, and hub genes were obtained by screening the degree of connectivity of each node in the network. In the network, nodes represent genes and lines represent the interactions. To identify the significant modules in the network, Cytoscape plug-in MCODE (Molecular Complex Detection) was conducted with a score > 4.

#### LncRNA and circRNA associated ceRNA network construction

According to the ceRNA hypothesis, ceRNA members can compete for the same miRNA response elements (MREs) to regulate each other. Various types of RNA transcripts compete with one another for binding to a shared miRNA, thereby negatively regulating miRNA-mediated gene silencing [23]. Hence, the expression pattern of mRNAs should be in the same direction as lncRNAs or circRNAs. The potential miRNA-binding sites were searched by the sequences of lncRNAs, circRNAs and mRNAs based on the TargetScan database (http://www.targetscan.org/). Then, lncRNA-miRNA-mRNA and circRNA-miRNA-mRNA networks were constructed based on lncRNA/circRNA-miRNA and miRNA-mRNA regulation pairs. In addition to overlapping miRNA binding, mRNAs with an expression pattern in the same direction as lncRNAs or circRNAs were filtered out to construct lncRNA/circRNA -miRNA-mRNA ceRNA networks by Cytoscape.

#### Quantitative real-time PCR assay

To validate our microarray data, quantitative Real-time experiments were performed as previously described [24]. We selected 13 representative RNAs to verify the expression, and primers sequences were listed in Table 2.

**Table 2:** Sequences of the primers used for PCR

Genes	Forward primer	Reverse primer
Cdc14a	TGCACTACACCTCTTTCGACC	AGGAGGGTTTGAGCCAGACA
Cnr2	ATGGCCGTGCTCTATATTATCCT	ATGGTCACACTGCCGATCTTC
Cd28	CTGTTCTTGGCTCTCAACTTCTT	GCTGACCTCGTTGCTATCTACC
Il18r1	ACTACTCCTGCGTGTTTTCTGTC	CATCCTTTCCTAGTTCTACACCAAC
Klrc1	CTCGCAGCTCCATTTCAGTC	CAATTAAGACAAAACAGATGAGGC
Ncr1	ATGCTGCCAACACTCACTG	ATGATGGGTTTCGGGAGAGTC
Ccl8	TCTACGCAGTGCTTCTTTGCC	AAGGGGGATCTTCAGCTTTAGTA
Rian	GGTGCTGCCTCAGTCTTT	TCCAGGATTGATTGTGCT
Foxd2os	ATCGGTAGTGGAAATCTGTAAG	TCAAAGCGACTGTATTAGGC
Snhg1	TCATGTTGTCACAGCACC	GCCCTTTACACTTTGGAG
Circ_0007379	AGACTGCCAGCCCCTCATC	GGGAAGCACTCTGGATGTTAGG
Circ_0004572	CATCAATGAAGTCAAGCCTACAGAG	CATGGACCTCGTACCCTTTCTC
GAPDH	GTGCCGCCTGGAGAAACC	GGTGGAAGAGTGGGAGTTGC

### Statistical analysis

Data were expressed as mean ± SD and were analyzed by one-way analysis of variance by SPSS version 18.0. *P* < 0.05 was considered significant different.

## Results

### Phenotypic characteristics

**Fig. 1:**
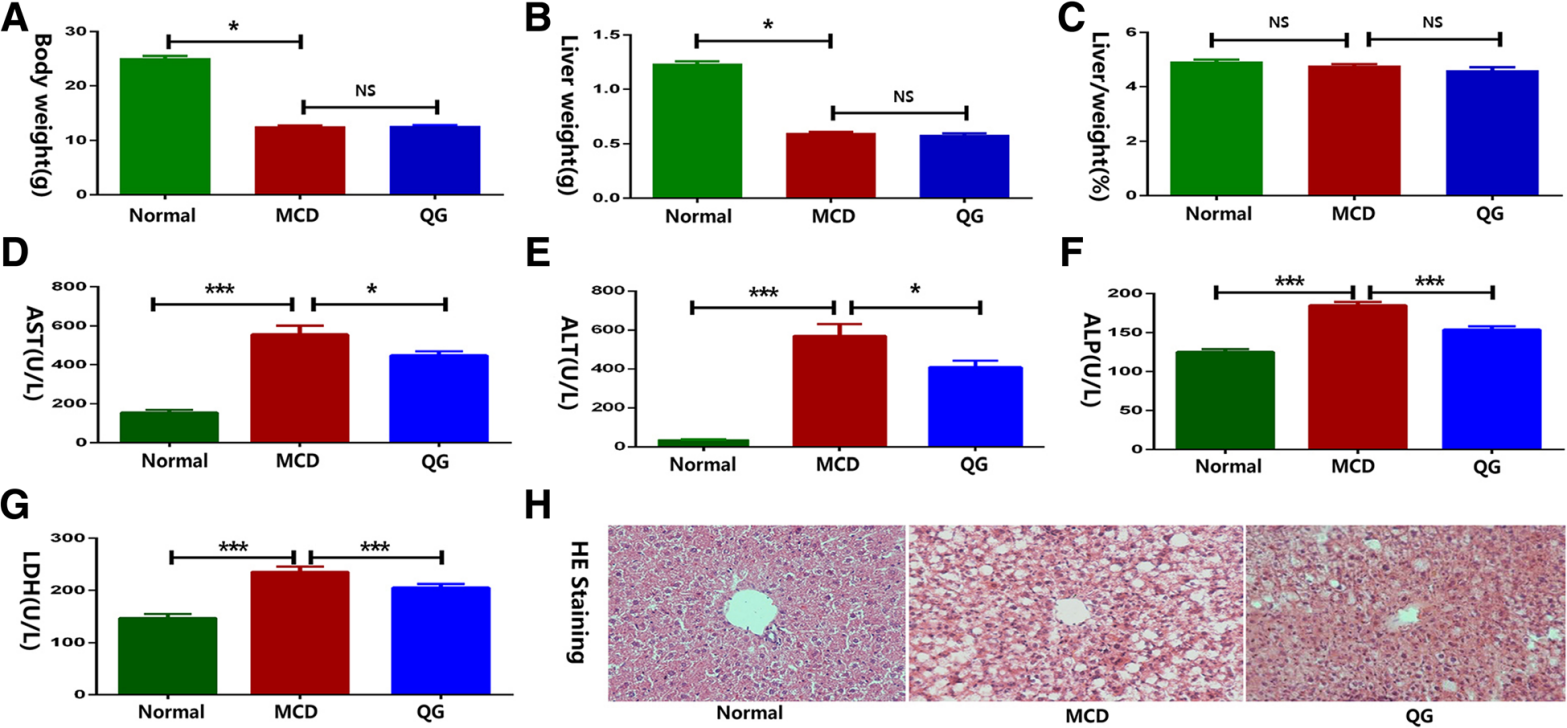
Phenotypic characteristics of the mice. **a** Body weight. **b** Liver weight. **c** Liver/body weight ratio. **d** Serum AST. **e** Serum ALT. **f** Serum ALP. **g** Serum LDH. **h** Liver sections stained with H&E. Data were represented as mean ± SD, *n* = 12 per group, ^a^ *P* < 0.05, ^b^ *P* < 0.01

Four weeks of MCD feeding induced typical NASH features, and H&E staining showed significant hepatic steatosis and inflammation in MCD mice compared to normal mice (Fig. 1h). Serum AST (Fig. 1d), ALT (Fig. 1e), ALP (Fig. 1f) and LDH (Fig. 1g) levels were all markedly increased in MCD mice. QG extract supplementation improved hepatic steatosis and inflammation, and restored increased serum parameters (Fig. 1d-g). The body weight and liver weight were decreased in the MCD mice in comparison to normal mice, while QG had no effect on these parameters (Fig. 1a&b). The ratio of liver weight and body weight among the groups revealed no significant difference (Fig. 1c).

### Differentially expressed mRNAs, lncRNAs and circRNAs

**Fig. 2:**
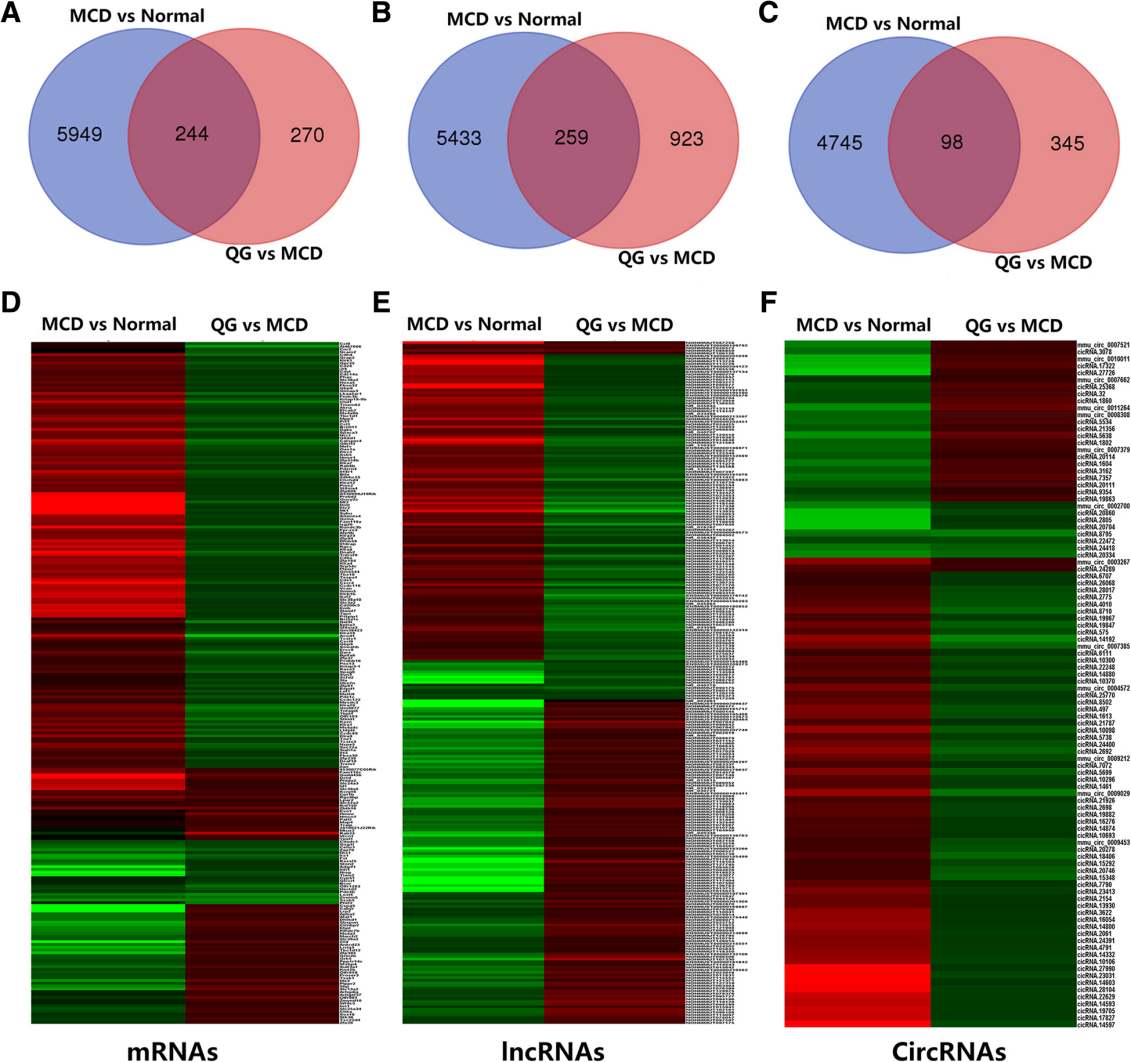
Hierarchical cluster of overlapped DEGs, DElncRNAs and DEcircRNA. **a** Venn diagram of overlapping DEGs. **b** Venn diagram of overlapping DElncRNAs. **c** Venn diagram of overlapping DEcircRNAs. **d** Heatmap of overlapping DEGs. **e** Heatmap of overlapping DElncRNAs. **f** Heatmap of overlapping DEcircRNAs. Gene expression is represented by colors, with brighter red for higher values and brighter green for lower values

By the cutoff of a 1.5-fold change and *P* < 0.05, 6193 differentially expressed mRNAs (differentially expressed genes, DEGs), 5692 differentially expressed lncRNAs (DElncRNAs) and 4843 differentially expressed circRNAs (DEcircRNAs) were identified in the MCD vs normal group. There were 514 DEGs, 1182 DElncRNAs and 443DEcicRNAs in QG vs MCD group. By Venn diagram, overlapping 244 DEGs (Fig. 2a), 259 DElncRNAs (Fig. 2b) and 98DEcircRNAs (Fig. 2c) were filtered out and chosen for further analysis. Representative overlapping DEGs, DElncRNAs and DEcircRNAs are listed in Tables 3, 4 and 5, respectively. Detailed information of differentially expressed RNAs was listed in Additional file 1: Table S1, Additional file 2: Table S2 and Additional file 3: Table S3.

**Table 3:** Representative overlapped differentially expressed genes

GeneSymbol	MCD vs Con Fold change	MCD vs Con *P*-value	QG vs MCD Fold change	QG vs MCD *P*-value
*Ccl8*	2.44	0.03	0.25	0.03
*Ssxb5*	0.31	0.00	0.33	0.01
*Cd28*	2.10	0.00	0.59	0.02
*Gm8677*	2.76	0.01	0.36	0.00
*Chil1*	4.73	0.03	0.36	0.02
*Catsper4*	5.35	0.04	0.37	0.02
*Stkld1*	2.61	0.01	0.37	0.02
*Klrk1*	2.15	0.04	0.59	0.02
*Olfr1283*	0.60	0.03	0.38	0.01
*Cdh4*	3.42	0.04	0.41	0.00
*Left1*	2.04	0.03	0.50	0.01
*Cd8a*	3.15	0.01	0.65	0.04
*Cabyr*	0.09	0.00	1.73	0.01
*Cxcl9*	2.09	0.00	0.64	0.15
*Il15*	1.62	0.02	0.65	0.02
*Il18r1*	4.44	0.05	0.46	0.05
*Sbp*	0.34	0.04	2.02	0.05
*Rgs9bp*	2.78	0.04	2.12	0.02
*Gadd45b*	9.64	0.04	2.22	0.03
*Zbtb16*	2.69	0.01	2.58	0.02

**Table 4:** Representative differentially expressed lncRNAs

GeneSymbol	MCD vs Con fold change	MCD vs Con *P*-value	QG vs MCD fold change	QG vs MCD P-value
*Snhg1*	2.47	0.02	0.63	0.00
*Slc36a3os*	4.24	0.04	0.35	0.03
*RP24-175C20.19*	0.61	0.05	1.62	0.01
*RP23-62D21.4*	0.49	0.00	1.98	0.00
*RP23-368 K22.5*	2.31	0.04	0.61	0.05
*Rian*	0.09	0.02	2.47	0.00
*Gm45609*	0.03	0.00	1.74	0.00
*Gm45414*	0.43	0.00	1.84	0.01
*Gm45204*	0.24	0.01	1.97	0.05
*Gm44427*	3.51	0.02	0.65	0.00
*Gm43823*	0.51	0.03	1.97	0.04
*Gm43814*	2.39	0.01	0.63	0.05
*Gm43168*	4.24	0.00	0.65	0.04
*Gm43065*	2.19	0.02	0.55	0.01
*Gm42640*	1.81	0.02	1.99	0.02
*Gm42599*	0.46	0.01	2.26	0.05
*Gm38351*	0.43	0.02	1.55	0.02
*Gm37570*	0.35	0.01	1.91	0.04
*Gm3716*	1.73	0.04	0.48	0.01
*Gm37143*	2.92	0.01	0.57	0.01
*Gm28875*	0.55	0.01	0.66	0.02
*Gm26645*	2.53	0.05	0.52	0.03
*Gm25694*	0.56	0.02	1.66	0.04
*Gm25545*	0.26	0.01	2.01	0.02
*Gm20633*	1.98	0.04	0.28	0.05
*Gm20052*	2.39	0.01	0.60	0.02

**Table 5:** Representative overlapped differentially expressed circRNAs

circBase_ID	MCD vs Con fold change	MCD vs Con *P*-value	QG vs MCD fold change	QG vs MCD *P*-value
circ_0011264	0.32	0.01	2.22	0.02
circ_0010011	0.24	0.00	1.80	0.03
circ_0009453	1.64	0.01	0.53	0.01
circ_0009212	2.35	0.04	0.54	0.02
circ_0009029	3.56	0.02	0.38	0.02
circ_0008308	0.44	0.03	1.76	0.02
circ_0007662	0.64	0.01	1.54	0.00
circ_0007521	0.35	0.01	1.68	0.03
circ_0007385	2.59	0.01	0.45	0.01
circ_0007379	0.43	0.03	1.68	0.03
circ_0004572	2.69	0.03	0.52	0.05
circ_0003267	3.11	0.01	1.56	0.04
circ_0002700	0.34	0.00	0.66	0.03

To obtain an overview of the expression pattern of DEGs, DElncRNAs and DEcircRNAs among groups, hierarchical clustering was performed. Most of the DEGs, DElncRNAs and DEcircRNAs exhibited opposite expression patterns between MCD vs normal and QG vs MCD (Fig. 2d-f). For instance, the DEGs *Cxcl9*, *Cd28*, *Cd8a*, *Btla*, *Klra1*, *Klrk1 and Ncr1*were up-regulated in the MCD vs normal group, but down-regulated in the QG vs MCD group. The DElncRNAs *Snhg1* and *Slc36a3os* were increased in the MCD vs normal group, but decreased in the QG vs MCD group. DEcircRNAscirc_0009029 and circ_0004572 were induced in the MCD vs normal group, but reduced in the QG vs MCD group. These results indicated that Qianggan extract could affect the gene expression of NASH mice.

### GO and pathway analysis

**Fig. 3:**
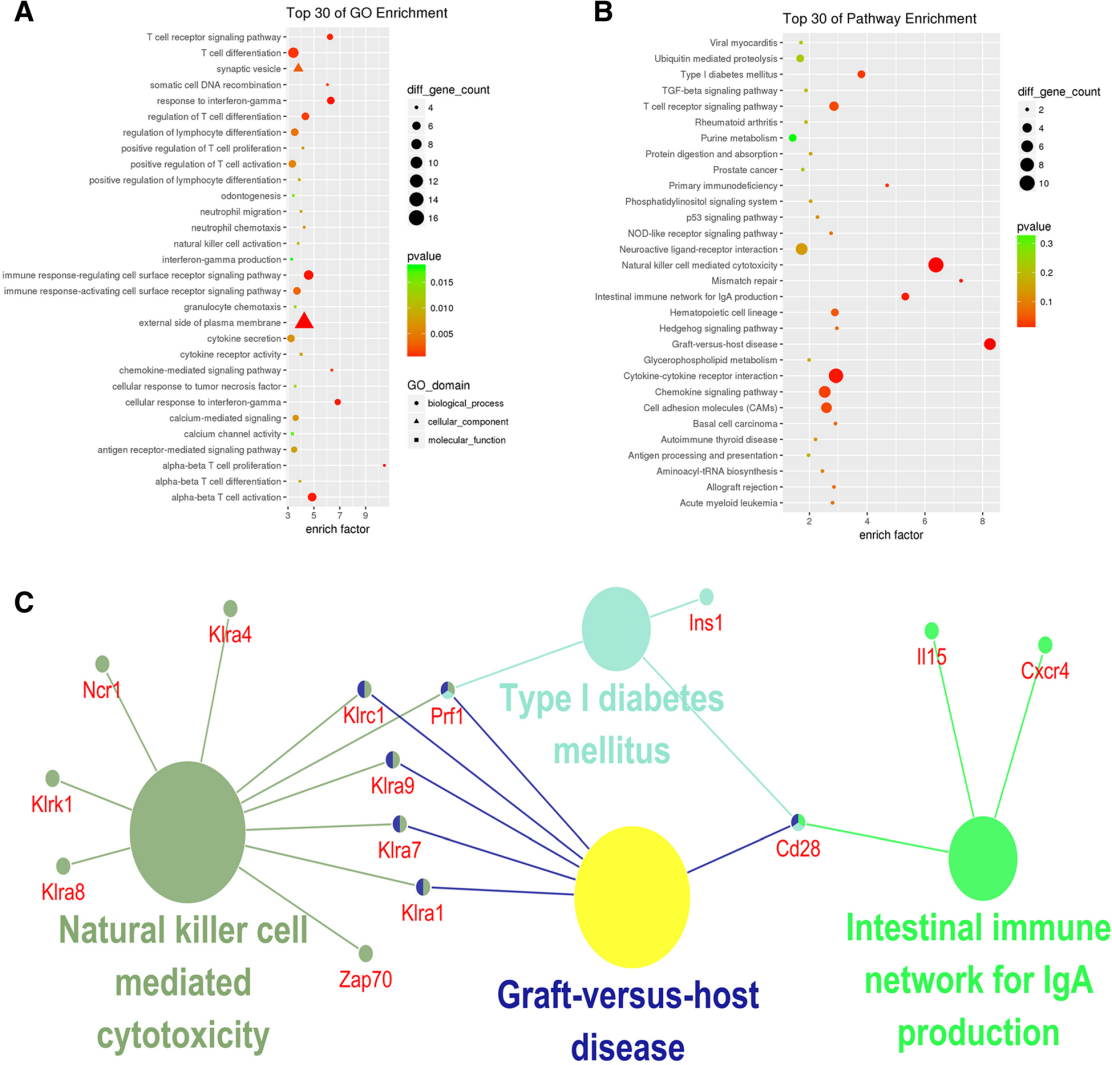
Functional enrichment of overlapping DEGs. **a** Top 30 enriched GO terms, y-axis represents GO terms, and x-axis represents enrichment factor. Size and color of the bubble represents amount of DEGs enriched in GO terms and enrichment significance, respectively. **b** Top 30 enriched KEGG pathways, y-axis label represents pathway names, and x-axis represents enrichment factor. Size and color of the bubble represents amount of DEGs enriched in the pathway and enrichment significance, respectively. **c** Pathway interaction, each node is a KEGG pathway item; the node size reflects pathway significance, edge between nodes reflected common genes; and different node colors represented different functional groups

To further explore the main biological functions of DEGs, GO and pathway enrichments were performed (Additional file 4: Table S4). The 244 overlapping DEGs were enriched in immune-related GO terms and KEGG pathways such as natural killer cell-mediated cytotoxicity, intestinal immune network for IgA production, and T-cell receptor signaling pathways. Figure 3a and b lists the top 30 enriched GO terms and KEGG pathways. To identify the key genes in the pathways, enriched KEGG pathway interactions were constructed, and hub genes such as *Cd28*, *Klra1*, and *Klra7* were indicated (Fig. 3c).

### PPI network analysis

**Fig. 4:**
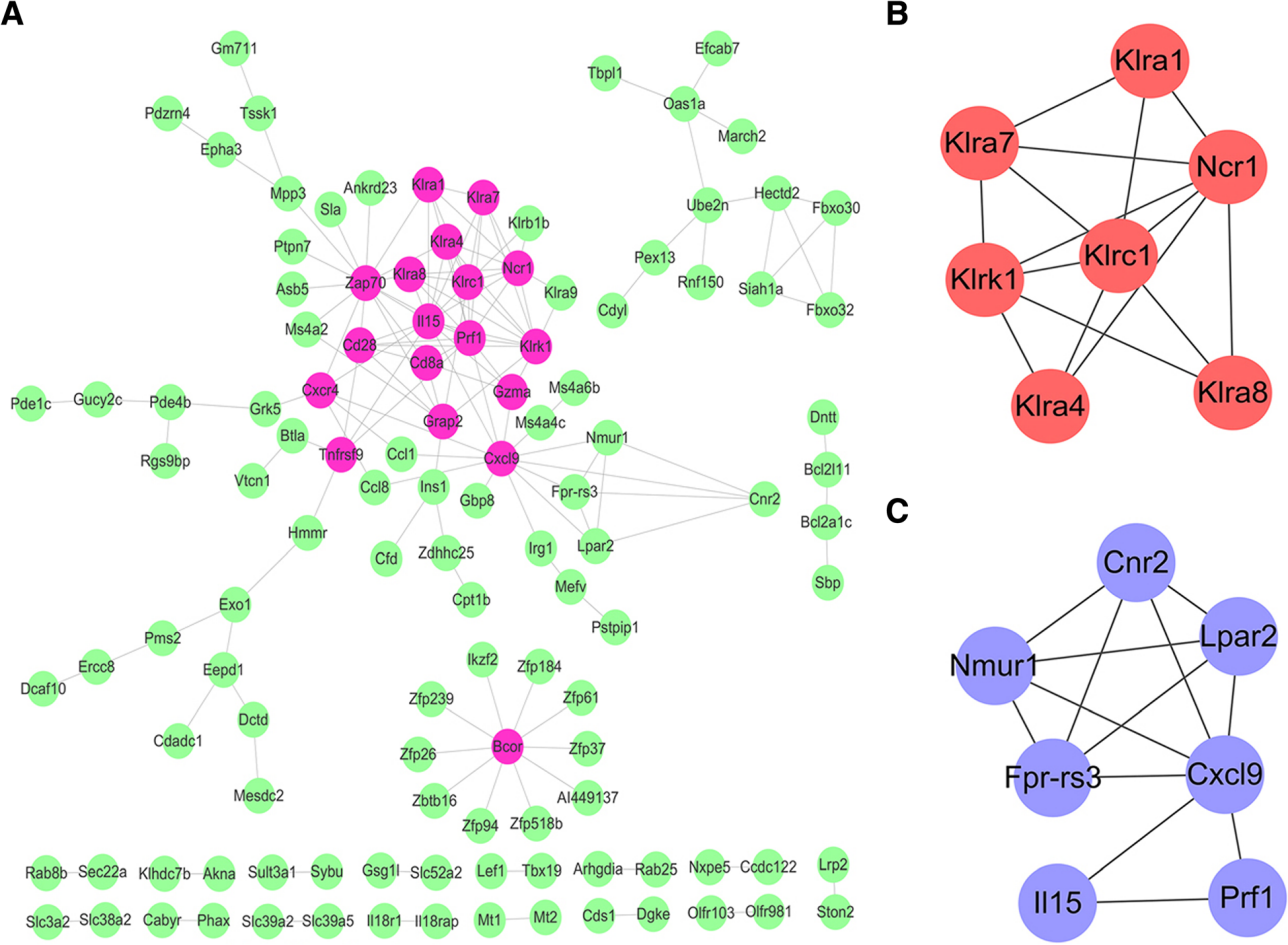
PPI networks. **a** PPI network with 118 nodes. Pink nodes represent the hub nodes with more neighbors (≥5). **b** A significant module with 7 nodes are identified by MCODE (score = 5). **c** A significant module with 7 nodes are identified by MCODE (score = 4.3)

PPI network analysis and MCODE were conducted to depict interactions and significant modules among the overlapped 244 DEGs. A PPI network with 118 nodes (average of 2.66 neighbors) was obtained (Fig. 4a). The important hub genes with no less than five neighbors were identified. By MCODE, two significant modules were filtered out with a score > 4. Most hub genes, such as *Cd28, Cd8a, Cxcl9, Klka1, Klrk1*and *Ncr1c*, and the modules were immune related.

### LncRNA and circRNA associated immune ceRNA networks

LncRNAs and circRNAs interacting as ceRNAs play important roles in regulating gene expression. However, the biological functions of most lncRNAs and circRNAs are unknown. To unravel the functions of identified DElncRNAs and DEcircRNAs, we constructed ceRNA networks. Interactions of miRNAs with mRNAs, lncRNAs and circRNA were predicted based on the Targetscan database (Additional file 5: Table S5). By combining of literature retrieval and the ceRNA hypothesis, we constructed two lncRNA- and four circRNA-associated immune ceRNA networks that were implied in NASH.

**Fig. 5:**
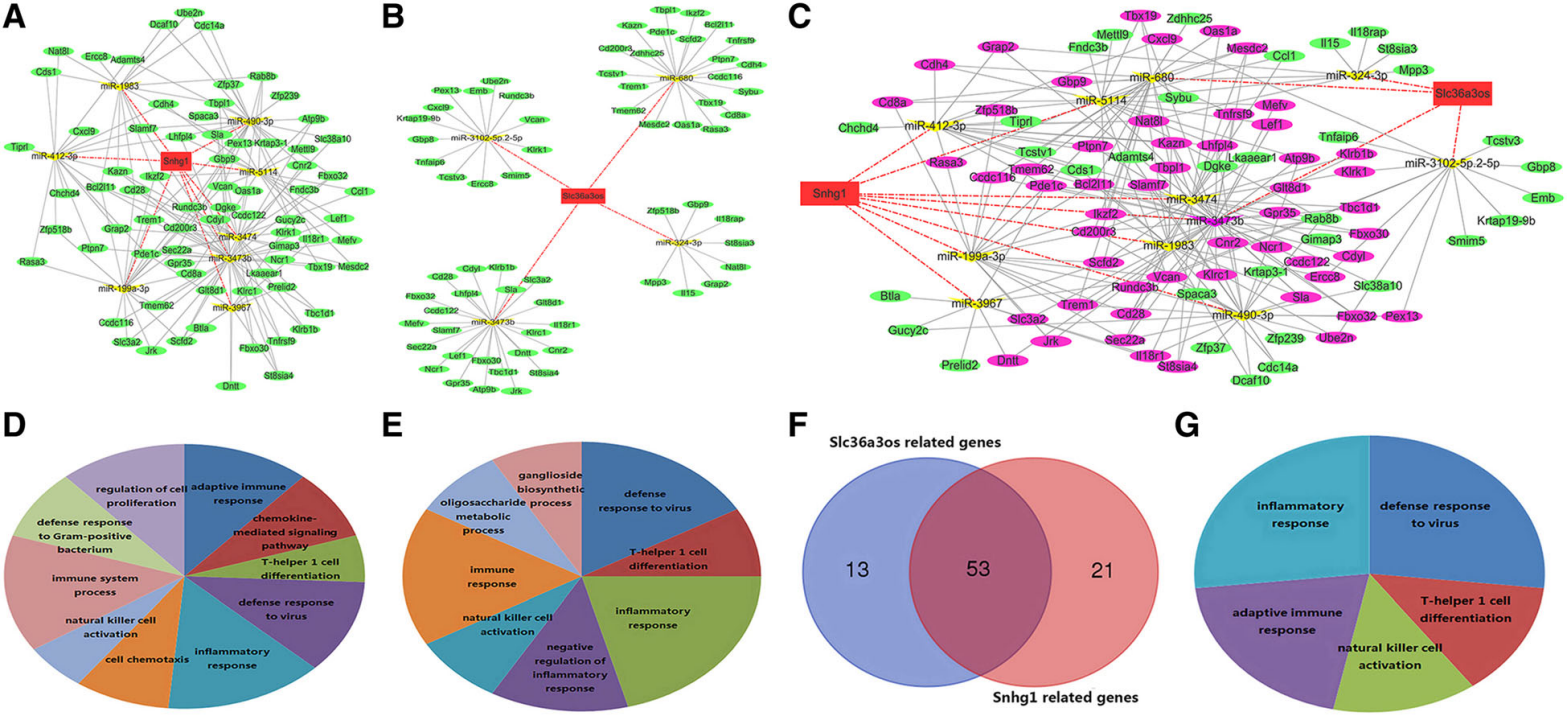
LncRNAs associated ceRNA networks. Red rectangles represent lncRNAs, green ellipses represent mRNAs, yellow triangles representsmiRNAs, and pink nodes representoverlapped RNAs. **a** lncRNAsnhg1 associated ceRNA networks. **b** lncRNASlc36a3os-associatedceRNA networks. **c** Merged ceRNA networks of lncRNAsnhg1and Slc36a3os. **d** Enriched GO terms of mRNAs involved in lncRNAsnhg1ceRNA network. **e** Enriched GO terms of mRNAs involved in lncRNASlc36a3osceRNA network. **f** Venn diagram of overlapped mRNAs between lncRNAsnhg1and Slc36a3os associated ceRNAs. **g** Enriched GO terms of overlapped mRNAs

LncRNA *Snhg1* was a ceRNA of eight miRNAs (e.g., miR-199a-3p, miR-490-3p, and miR-412-3p) that targeted 74 mRNAs (e.g., *Cd28, Cd8a*, and *Cxcl9*) (Fig. 5a). LncRNA *Slc36a3os* was a ceRNA of four miRNAs (miR-324-3p, miR-680, miR-3473b and miR-3102-5p.2-5p) that targeted 66 mRNAs (e.g., *Cd28, Il18rap,* and *Il15*) (Fig. 5b). LncRNA *Snhg1* and *Slc36a3os* shared 53 mRNAs such as *Cd28, Cd8a,* and *Klrk1* (Fig. 5c &f). Moreover, the GO terms revealed that mRNAs involved in lncRNA*Sngh1* or *Slc36a3os* ceRNA networks were enriched in immune processes (Fig. 5d, e&g).

**Fig. 6:**
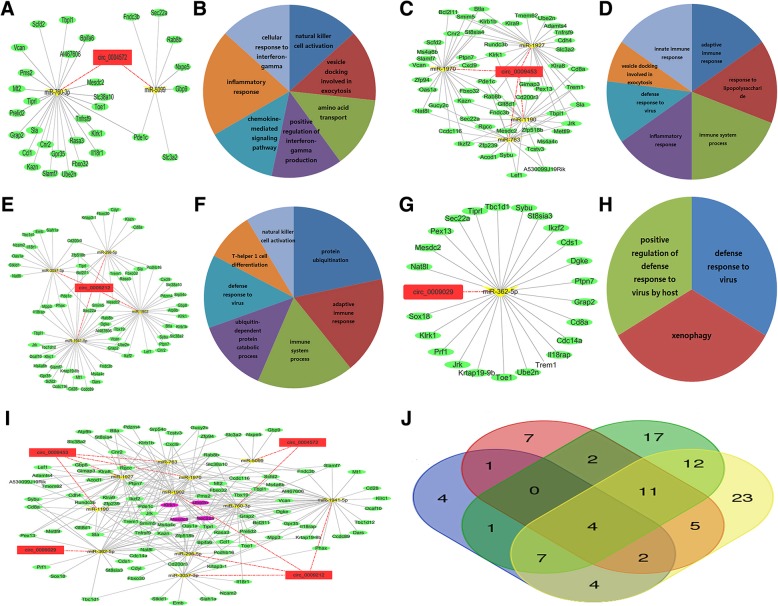
CircRNA-associated ceRNA networks. Red rectangles represent circRNAs, green ellipses represent mRNAs, yellow triangles represent miRNAs, and pink nodes represent overlapped RNAs. **a** circ_0004572-associated ceRNA networks. **b** Enriched GO terms of mRNAs involved in circ_0004572 networks. **c** circ_0009453 associated ceRNA networks. **d** Enriched GO terms of mRNAs involved in circ_0009453 network (**e**) circ_0009212-associated ceRNA networks. **f** Enriched GO terms of mRNAs involved in circ_0009212 network. **g** circ_0009029-associated ceRNA networks. **h** Enriched GO terms of mRNAs involved in circ_0009029 network. **i** Merged ceRNA networks of four circRNAs. **j** Venn diagram of overlapped mRNAs among four circRNA-associated ceRNAs. Blue represents circ_0009029-associated mRNAs, red represents circ_0004572-associated mRNAs, green represents circ_0009453-associated mRNAs and yellow represents circ_0009212-associated mRNAs

In addition, circ_0004572 was a ceRNA of 2 miRNAs that targeted 32 mRNAs (Fig. 6a). Circ_0009453 was a ceRNA of four miRNAs that targeted 54 mRNAs (Fig. 6c)*.* Circ_0009212 was a ceRNA of 4 miRNAs that targeted 68 mRNAs (Fig. 6e)*.* Circ_0009029 was a ceRNA of miR-362-5p that targeted 24 mRNAs (Fig. 6g). GO analysis demonstrated that mRNAs involved in the networks were enriched in immune-related terms (Fig. 6b, d, f and h). We obtained circRNA-associated immune ceRNA networks that comprised 4 circRNAs, 11 miRNAs and 100 m RNAs, and we also noticed that the four circRNAs shared four mRNAs (Fig. 6i and j).

### Validation of the RNA expression

**Fig. 7:**
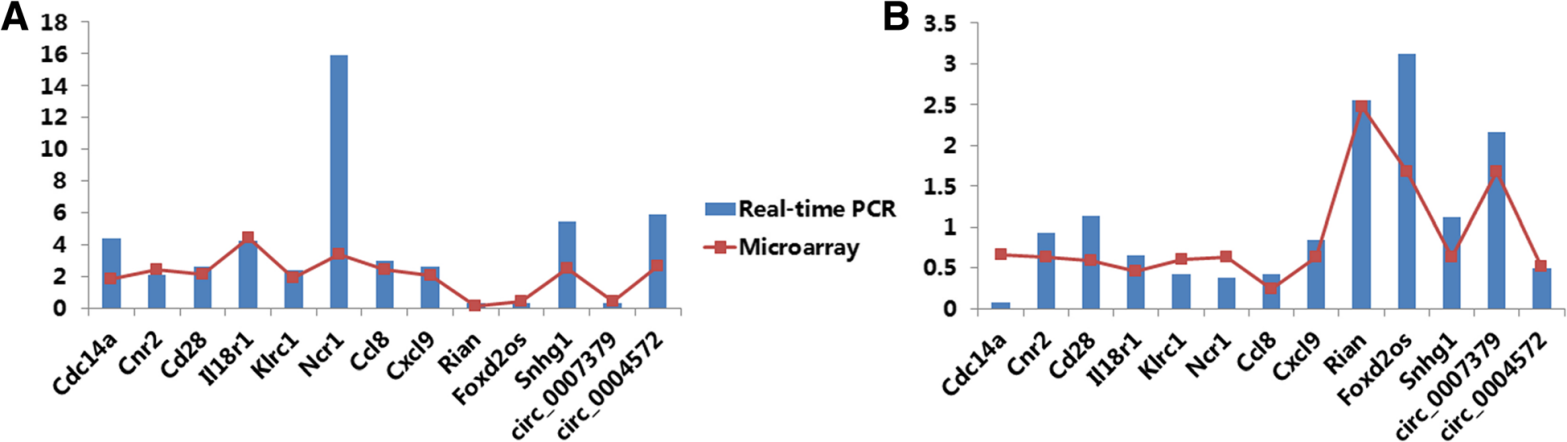
Validation of representative RNAs. The x-axis represents RNA names, and the y-axis represents RNA fold changes based on average expression values. Blue bars represent data yielded by real-time qPCR, and red line with points represent data obtained by microarray. **a** MCD vs normal groups, (**b**) QG vs MCD groups

To validate our findings based on microarray data, we selected 13 RNAs for verification by RT-qPCR. Fold changes of RNAs (MCD vs normal and QG vs MCD) were calculated based on the average expression values. As shown in Fig. 7, most of the results from RT-qPCR were in consistence with microarray data, which might suggest the reliability of our microarray analysis and findings.

## Discussion

Here we confirmed the effect of QG on NASH mice, and comprehensive microarray analysis revealed a batch of differentially expressed mRNAs, lncRNAs and circRNAs. Through bioinformatics analysis, we constructed lncRNA- circRNA-associated immune ceRNA networks, and obtained certain immune-related biological processes, pathways and hub genes that responded QG intervention.

Qianggan formula was initially designed for treat hepatitis B virus in China, and it showed obvious effect in reducing plasma ALT, AST and inflammatory parameters [25]. In clinical trials of NAFLD, Qianggan formula treatment significantly reduced hepatic lipid content in patients, as evidenced by ultrasound test. In addition, plasma TG and ALT levels were decreased upon treatment [26]. In high-fat diet-induced NAFLD rats, 4 weeks of QG treatment prevented leptin resistance, and alleviated lipid accumulation and inflammation in the liver [27]. Because the formula is a mixture of 16 ingredients and undoubtedly has multiple targets, integrative high-throughput analysis could facilitate the exploration of the underlying mechanisms, and the ceRNA networks we have reported may provide valuable information.

Immune responses are involved in the development of NASH [28]. Many types of innate (NK cells, NKT cells, and kupffer cells/macrophages) and adaptive (T cells and B cells) immune cells are enriched in the liver and play pivotal roles in NASH progression [29]. NK- cell-mediated cytotoxicity and the T-cell receptor signaling pathway are important immune processes, and studies have shown that the dysfunction of these pathways is involved in liver inflammation. Accordingly, we identified changes in several immune hub genes, such as *Cd28*, *Cd8a*, *Cxcl9*, *Klra1*, *Klrk1*, *Klra9*, *Il15*, and *Ncr1*that related to the effect of Qianggan extract.

LncRNAs might have important roles in NAFLD/NASH [30]. LncRNA *SNHG1* is up-regulated by *MYCN* amplification and could be a potential prognostic biomarker for neuroblastoma intervention in patients [31]. Another study reported that decreased lncRNA*SNHG1* inhibits colorectal carcinoma tumorigenesis and might act as an important potential therapeutic target [32]. Of note, lncRNA *Snhg1* is reported to be increased in hepatocellular carcinoma [33]. In the present study, we observed that the level of lncRNA*Snhg1*was increased in NASH, whereas it was decreased by Qianggan extract. Considering the association between NASH and hepatocellular carcinoma, lncRNA*Snhg1* might be a therapeutic target of QGE and a potential biomarker of NASH.

According to the ceRNA hypothesis, lncRNAs act as miRNA sponges to negatively regulate miRNAs and positively regulate target mRNAs. A recent study revealed that lncRNA *SNHG1* acted as a ceRNA of miR-326 targeted NOB1 [34]. A previous report showed that lncRNA *SNHG1* serves as a ceRNA to negatively regulate miR-199a-3p and enhance *CDK7* expression, which might be a potential therapeutic target for prostate cancer [35]. We noticed that lncRNA *Snhg1* serves as a ceRNA of miR-199a-3p, and 7 other miRNAs that targeted a series of immune-related mRNAs. CD28 is a membrane protein belonging to the family of co-stimulatory molecules that synergistically acts as a second signal with the T-cell receptor complex [36]. Researchers have revealed that deletion of CD28 establishes a new pro/anti-inflammatory balance, and protects the liver against steatosis [37]. In accordance with previous studies, we observed that *Cd28* mRNA expression was elevated in NASH and reduced after QGE treatment in mice. CD8A is a constituent of the CD8 antigen found on most cytotoxic T cells and acts as a co-receptor with the T-cell receptor [38]. Here, we noticed that *Cd8a* was up-regulated in NASH mice but down-regulated after Qianggan treatment, suggesting *Cd8a* as a potential therapeutic target for NASH. CXCL9 is secreted by monocyte-derived dendritic cells within the liver and is a pro-inflammatory cytokine that attracts Th1 and Th17 cells to the liver [39]. CXCL9 was elevated in patients with autoimmune hepatitis and mice with fatty liver [40, 41], indicating that CXCL9 inhibition is a potential strategy in NAFLD treatment.

MiR-324-3p is associated with malignant clinicopathologic features and poor prognosis of hepatocellular carcinoma [42]. In the present study, we noticed that miR-324-3p-targeted mRNAs were differentially expressed among the groups. IL-15 has been implicated in high-fat-diet-induced hepatic lipid accumulation and inflammation through immune regulation [43]. Researchers have analyzed the level of IL-15 in patients with acute hepatic failure and have suggested that overexpression of IL-15 might cause liver injury in humans [44]. Similar to lncRNAs, circRNAs can also act as ceRNAs to inhibit miRNAs and positively regulate associated mRNAs. We identified several circRNAs acting as ceRNAs in NASH. MiR-760-3p has been implicated in immunological processes and miR-5099 in liver injury [45, 46]. Intriguingly, we observed that 32 targeted mRNAs were enriched in immune related functions and several mRNAs (e.g., *Ccl1* and *Il18r1)* were related to liver inflammation and fibrosis. Inhibition of the CC chemokine ligand CCL1 has been suggested as a successful therapeutic way to limit liver inflammation and fibrosis [47]. In addition, IL18R1^−/−^ mice displayed a less severe liver fibrotic phenotype than wild-type animals [48].

*Klrk1*, also known as *Nkg2d*, is an activating receptor expressed on the surface of NK cells, CD8^+^ T cells, and subsets of CD4^+^T cells and invariant NKT cells [49]. *KLRK1* mRNA increased in patients with NASH [50]. Lymphoid enhancer binding factor *Lef1* is essential for the early stages of thymocyte maturation, and is involved in liver fibrosis [51, 52]. The physiological significance of miR-362-5p in the regulation of NK cell function has been studied [53]. Up-regulation of miR-362-5p is associated with hepatocellular carcinoma and inhibition of miR-362-5p dramatically decreases tumor growth and metastasis [54]. Regulation of miR-362-5p could be inferred from our results. MiR-296-5p expression is reduced in liver samples from NASH patients [55]. Here, we observed that cir_0009212 and miR-296-5ptargeted mRNAs were increased in NASH mice and that QG partially restored these alterations.

Although we obtained abundant information through ceRNA networks, and hinted at the molecular mechanisms of QG on NASH, the limitations of our study should also be noted. The MCD diet-induced mice manifested obvious hepatic steatosis and inflammation, but lacked the human metabolic profile, such as insulin resistance, and dyslipidemia. The substantial weight loss of the mice was not observed in NASH patients. In addition, since we only investigated the levels of mRNAs and not their coded proteins, a thorough and comprehensive human investigation needs to be performed to confirm the results.

## Conclusions

Our comprehensive expression profiles of mRNAs, lncRNAs and circRNAs revealed that QG might ameliorate NASH by modulating lncRNA and circRNA associated ceRNA networks. This may broaden our knowledge of QG targets and have promising therapeutic implications for NASH.

## Additional files

Additional file 1: Detailed information of DEGs among groups. Sheet1: The 6193 DEGs in MCD vs normal group. Sheet2: The 514 DEGs in in QG vs MCD group. Sheet3: The overlapped 244 DEGs between MCD vs normal and QG vs MCD. (XLSX 545 kb)

Additional file 2: Detailed information of DElncRNAs among groups. Sheet1: The 5692 DElncRNAs in MCD vs normal group. Sheet2: The 1182 DElncRNAs in in QG vs MCD group. Sheet3: The overlapped 259 DElncRNAs between MCD vs normal and QG vs MCD. (XLSX 453 kb)

Additional file 3: Detailed information of DEcircRNAs among groups. Sheet1: The 4843 DEcircRNAs in MCD vs normal group. Sheet2: The 443 DEcircRNAs in in QG vs MCD group. Sheet3: The overlapped 98 DEcircRNAs between MCD vs normal and QG vs MCD. (XLSX 349 kb)

Additional file 4: Detailed information of GO germs and KEGG pathways. Sheet1: GO terms. Sheet2: KEGG pathways. (XLSX 64 kb)

Additional file 5: Detailed information of predicted miRNAs for DEGs, DElncRNAs and DEcircRNAs. Sheet1: The predicted miRNAs for DEGs. Sheet2: The predicted miRNAs for DElncRNAs. Sheet3: The predicted miRNAs for DEcircRNAs. (XLSX 771 kb)

## Data Availability

The datasets used and/or analyzed during the current study are available from the corresponding author on reasonable request.

## Abbreviations

ALP: Alkaline phosphatase
ALT: Serum alanine aminotransferase
AST: Aspartate aminotransferase
ceRNAs: Competing endogenous RNAs
circRNAs: Circular RNAs
DEcircRNAs: Differentially expressed genes circRNAs
DEGs: Differentially expressed genes
DElncRNAs: Differentially expressed lncRNAs
GO: Gene ontology
KEGG: Kyoto Encyclopedia of Genes and Genomes
LDH: Lactate dehydrogenase
lncRNAs: Long non-coding RNAs
MCD: Methionine-and choline-deficient diet
MCODE: Molecular Complex Detection
miRNAs: MicroRNAs
mRNAs: Ribonucleic acids
NAFLD: Nonalcoholic fatty liver disease
NASH: Non-alcoholic steatohepatitis
ncRNAs: Non-coding RNAs
PPI: Protein-protein interaction
QG: Qiang-Gan extracts
